# Dexamethasone enhances the lung metastasis of breast cancer via a PI3K-SGK1-CTGF pathway

**DOI:** 10.1038/s41388-021-01944-w

**Published:** 2021-07-16

**Authors:** Yujing Zhang, Gang Shi, Hantao Zhang, Qi Xiong, Fuyi Cheng, Huiling Wang, Jieyan Luo, Yong Zhang, Pengyi Shi, Jia Xu, Jiamei Fu, Na Chen, Lin Cheng, Yiming Li, Lei Dai, Yang Yang, Dechao Yu, Shuang Zhang, Hongxin Deng

**Affiliations:** 1 State Key Laboratory of Biotherapy and Cancer Center, West China Hospital, Sichuan University, Chengdu, PR China; 2 Department of Biotherapy, Cancer Center, West China Hospital, Sichuan University, Chengdu, Sichuan PR China; 3 Innovent Biologics, Inc, Suzhou, Jiangsu PR China; 4 Research Unit of Gene and Immunotherapy, Chinese Academy of Medical Sciences, Beijing, PR China

**Keywords:** Breast cancer, Prognostic markers

## Abstract

Dexamethasone (Dex), as a pretreatment agent, is widely used to attenuate the side effects of chemotherapy in breast cancer treatment. However, whether and how Dex affects breast cancer metastasis remain to be furtherly understood. In this study, we established several mouse breast cancer metastatic models to study the effect of Dex in vitro and in vivo. Transwell, Western Blot and RNA interference were applied to study the molecular mechanism of Dex in promoting breast cancer cell migration. Meanwhile, the effect of Dex on lung metastasis of breast cancer in Dex combined with PTX chemotherapy was discussed. Our results confirmed that Dex could promote breast cancer cell metastasis both in vitro and in vivo. Mechanistic studies revealed that this pro-metastatic effect of Dex was mediated by the GR-PI3K-SGK1-CTGF pathway in tumor cells. Ligation of Dex and glucocorticoid receptor (GR) on tumor cells activated the PI3K signaling pathway and upregulated serum glucocorticoid-inducible kinase 1 (SGK1) expression, and then increased the expression of connective tissue growth factor (CTGF) through Nedd4l-Smad2. Moreover, Dex was the leading factor for lung metastasis in a standard regimen for breast cancer treatment with paclitaxel and Dex. Importantly, targeting SGK1 with the inhibitor GSK650394 remarkably reduced lung metastasis in this regimen. Our present data provide new insights into Dex-induced breast cancer metastasis and indicate that SGK1 could be a candidate target for the treatment of breast cancer metastasis.

## Introduction

Malignant tumors are characterized by their ability to metastasize, and several discrete steps included cellular adhesion loss, increased migration ability, entry in the circulation, escape from immune surveillance, and eventual colonization of a distant site. In addition to individual genetic heterogeneity, the multiple drugs involved in treatment may be associated with cancer metastasis [1–3]. More recently, A growing body of literature has emerged, which offers contradictory finding that chemotherapy and adjuvant drugs exert cytotoxicity while increase cancer metastasis [4, 5]. However, our understanding of the potential molecular mechanisms and signaling pathways underlying these phenotypic changes is still fragmentary.

Glucocorticoid such as dexamethasone are among the most commonly prescribed immunosuppressive agent widely used in inflammatory diseases [6]. Dexamethasone is also commonly used to treat patients with cancer to combat the side effects of chemotherapy (such as nausea, vomit, edema, allergic reactions) and to treat symptoms related to advanced cancer. However, the effects of Dex are double edged, and considerable concerns have been expressed regarding the widespread use in solid tumors. It is well known that Dex induces significant generalized immunosuppression, which is associated with suppressed lymphocyte proliferation, impaired natural killer cells, and decreased lymphokine production [7–9]. Meanwhile, Dex could also affects cancer cell directly, which contributes to marked diversity in biological behavior of many solid cancers. Dex can increase apoptosis in lymphomas and other hematologic malignancies [10]. However, Dex increases glucocorticoid receptor (GR)-mediated reporter activity and cell proliferation while inhibiting apoptosis and cell invasion by suppressing the expression of MMP-2/ MMP-9, IL-6 and inducing mesenchymal-to-epithelial transition (MET) in human bladder cancer lines [11]. Furthermore, some clinical trials have been already performed to evaluate the impact of dexamethasone on the survival of cancer patients. According to the results of recent study, preoperative dexamethasone treatment was associated with higher rate of distant recurrence in colon cancer patients [12].

Breast cancer is the most common malignancies in women and the leading cause of death globally [13, 14]. Prior studies have suggested that the activation of the glucocorticoid receptor promoted breast cancer metastasis, which correlate with reduced survival in patient-derived xenograft models in mice [15]. Therefore, it is necessary to investigate whether and how dexamethasone, frequently used in the management of breast cancer, affects the overall survival and other outcomes, which addresses concerns about its direct effects against tumor cells and immunosuppressive properties.

Our research examined the emerging role of Dex in the context of breast cancer metastasis. We demonstrated that Dex could promote the migration of breast cancer cells through SGK1-CTGF signaling, and that ablation of SGK1 or CTGF reduced metastatic outgrowth and prolonged survival in mouse models. Our results aim to elucidate the mechanism of Dex in regulating breast cancer metastasis, which provides theoretical basis and experimental data for clinical strategy.

## Results

### Dex promotes lung metastasis of mouse 4T1 breast cancer

**Fig. 1:**
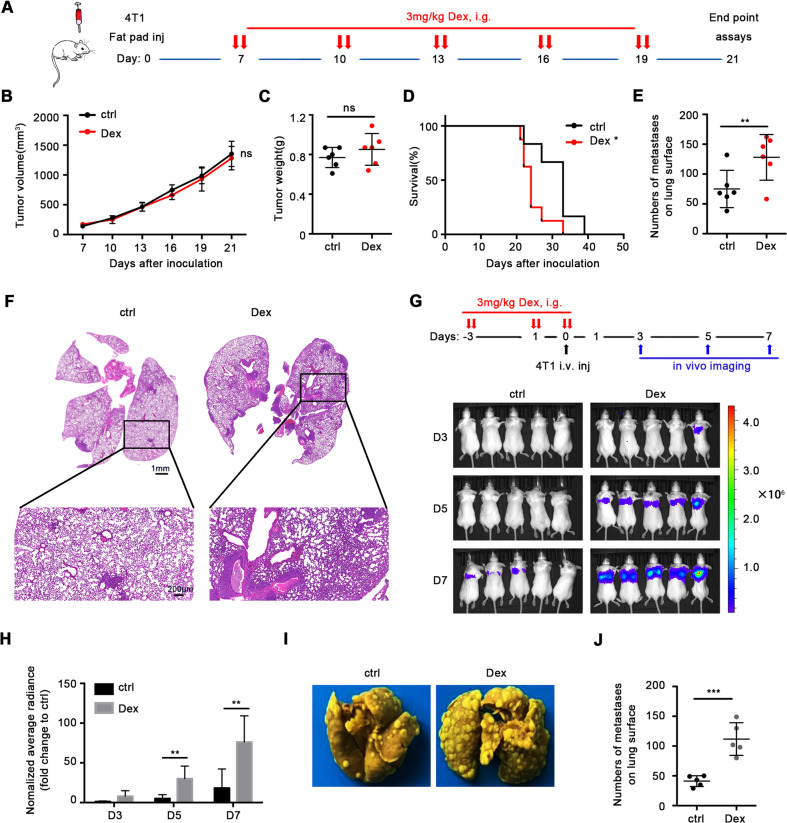
Dex exacerbates breast cancer lung metastasis in vivo. **A** Mice treatment scheme. **B** Tumor volume (*n* = 6 mice per group). **C** Tumor weight at the end point (*n* = 6 mice per group). **D** Overall survival (*n* = 10 mice per group). **E** Number of metastases on lung surface (*n* = 6 mice per group). **F** Typical H&E staining of lung. Mice were treated as scheme and lung metastasis was evaluated on day 3, 5, 7 after inoculation by in vivo bioluminescence (*n* = 5 mice per group), image of bioluminescence (**G**) and statistics of average radiance (**H**) were showed. **I** Typical mouse lung tissue fixed with Bouin’s fixation. **J** Number of metastases on lung surface in 4T1-luc experimental metastasis model (*n* = 5 mice per group). Mean ± SEM is shown. *p < 0.05, **p < 0.01, ***p < 0.001; ns no statistical significance.

We established the orthotopic 4T1 murine mammary carcinoma model to detect the effect of Dex. When tumor volumes reached 50–100 mm^3^, mice were treated with Dex following a schedule (Fig. 1A). The results showed that compared with the control group, Dex had no effects on either tumor growth or weight (Fig. 1B, C). However, mice treated with Dex demonstrated shorter survival (Fig. 1D). As cancer metastasis is a pivotal lethal factor, to verify this hypothesis, we investigated lung metastasis after Dex treatment. The results showed that administration of Dex remarkably increased the number of lung metastases (Fig. 1E). Further histological evaluation also confirmed these results (Fig. 1F).

In order to further investigate the pro-metastatic effects of Dex, we established experimental metastasis model by intravenous injection of 4T1-luc cells and treated mice following the schedule (Fig. 1G). The data showed that Dex treatment enhanced the implantation of tumor cells in the lung (Fig. 1G, H). A similar conclusion was also achieved through analysis of metastatic tumor nodes (Fig. 1I, J). Next, we asked if this effect was difference due to the species difference of tumor cells, a human breast cancer line, MDA-MB-231, was tested and the results suggested that Dex exhibited a similar pro-metastatic effect in a human breast cancer model (Supplementary Fig. S1A–C).

These results demonstrated that Dex can enhance metastasis and reduce overall survival in both experimental and orthotopic mouse models.

### Dex increases the migration ability of breast cancer cells

**Fig. 2:**
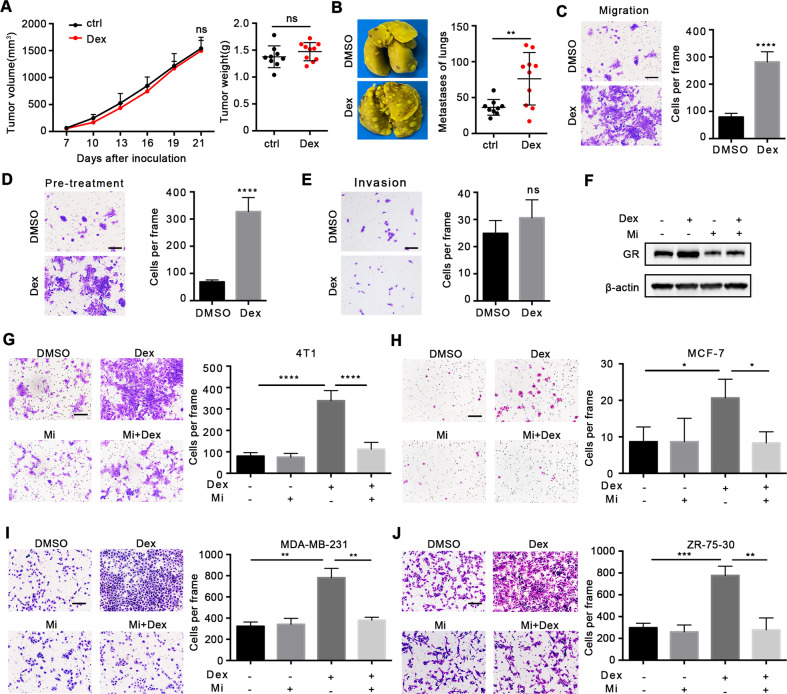
Dex directly promotes the migration of breast cancer cells. **A**, **B** Orthotopic 4T1 model in BABL/c nude mice (*n* = 9 or 10 mice per group). **A** Tumor growth (left) and tumor weight (right) were shown. **B** Metastases on lungs surfaces. **C**, **D** The migration assay of 4T1 cells in different condition. **C** 100 nM Dex was added in the lower chamber of Transwell or **D** 4T1 cells were pretreated with 100 nM Dex for 48 h and then were used for Transwell. **E** The invasion ability of 4T1 cells. **F** The GR expression of 4T1 cells treated with Dex/Mifepristone for 48 h. The influence of GR inhibitor Mifepristone on migration assay of 4T1 cells (**G**) and human breast cancer cells including MCF-7 (**H**), MDA-MB-231 (**I**), ZR-75-30(**J**). The scale bar is 25 μm. Mean ± SEM is shown. *p < 0.05, **p < 0.01, ***p < 0.001, ****p < 0.0001; ns no statistical significance.

Anti-inflammatory and immunosuppressive effects are two benefits of Dex treatment in clinical use [16]. Based on the important role of immune surveillance in inhibition of metastasis [17–19], we determined whether metastasis was caused by the immunosuppressive capacity of Dex. Results showed that Dex could still promote lung metastasis by 4T1 cells in immune-deficient mice (Fig. 2A, B), indicating that prometastatic effect was independent of immunosuppressive ability of Dex.

We proposed that Dex might directly affect tumor cells. To this end, we treated tumor cells with Dex in vitro. The results of Transwell assays showed that Dex exacerbated the migratory ability of 4T1 cells (Fig. 2C). To study whether persistent treatment was essential for the pro-metastatic effects of Dex, 4T1 cells were pre-treated with Dex for 48 h and then used for Transwell assays. The data suggested that 4T1 cells with pre-treatment also demonstrated stronger migration ability (Fig. 2D). Moreover, the invasive ability of 4T1 cell was not altered by Dex (Fig. 2E), and irrespective of estrogen receptor (ER) status, Dex treatment did not affect the proliferation and colony-forming capacities of breast cancer cells in the absence of estrogen stimulation (Supplementary Fig. S2A, B).

The biological effects of Dex are mediated through binding to GR [20–22], and treatment with the GR inhibitor mifepristone (MedChemExpress, New Jersey, USA) could effectively inhibit the expression of GR in 4T1 cells (Fig. 2F) and reduced tumor cell migration induced by Dex in both 4T1 cells (Fig. 2G) and human breast cancer cells (Fig. 2H–J). Taken together, these results indicated that Dex could directly promote breast cancer cell migration via GR signaling pathway.

### Dex promotes metastasis through the PI3K pathway

**Fig. 3:**
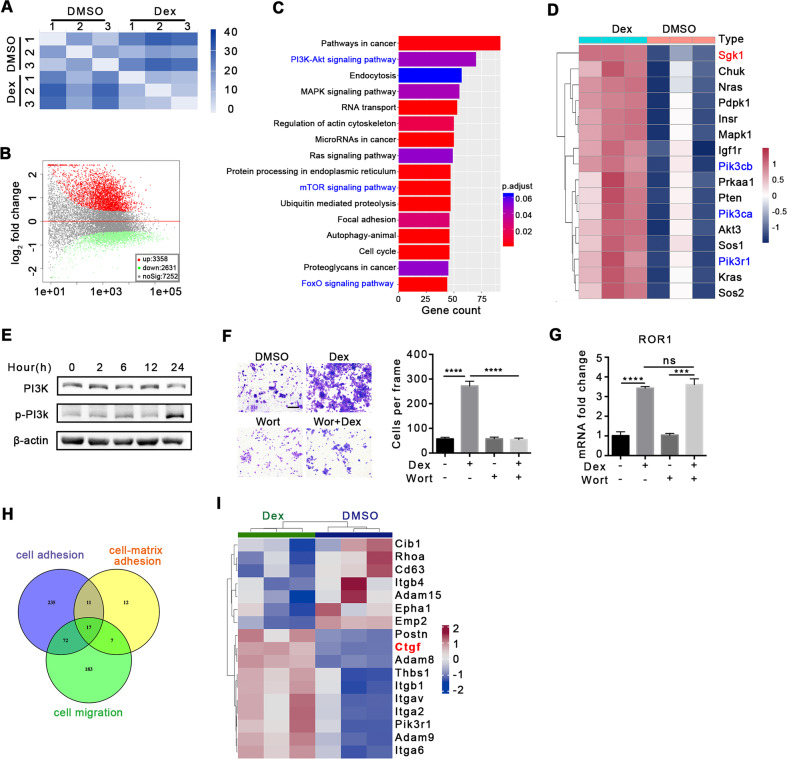
Transcriptome sequencing analysis of 4T1 cells post Dex treatment. **A** Sample-to-sample distances heatmap, *n* = 3 replicates for each group. **B** Volcano map of differential gene expression. **C** KEGG analysis of gene pathway. **D** Overlap genes among PI3K-Akt, mTOR and FoxO signaling pathways. **E** The activation of PI3K in 4T1 cells after Dex (100 nM) treatment. **F** The influence of PI3K inhibitor Wortmanin on the migration of 4T1 cells. The scale bar is 25 μm, *n* = 3 replicates. **G** The expression of ROR1 after Dex/ Wortmanin treatment for 48 h, *n* = 3 replicates. **H** Venn analysis of genes related to *cell adhesion*, *cell-matrix adhesion* and *cell migration*. **I** Heat map of differential genes related to adhesion. Mean ± SEM is shown. ***p < 0.001, ****p < 0.0001; ns no statistical significance.

Epithelial-mesenchymal transition (EMT) is a key step in tumor metastasis [23, 24]. Nevertheless, in our study, Dex did not alter the EMT process (Supplementary Fig. S3A, B), and we speculated that other mechanisms are involved in regulation of Dex-induced metastasis. To provide an overall insight into Dex-induced metastasis, transcriptome sequencing was performed in 4T1 cells treated with Dex. As shown in Fig. 3, matrix analysis between the two groups yielded distinct expression profile (Fig. 3A). A total of 5989 genes were screened based on a P-adjusted value of <0.05, which contained 3358 upregulated genes and 2631 downregulated genes (Fig. 3B). Furthermore, KEGG analysis with differentially expressed genes (*P* < 0.05) revealed that PI3K-Akt, mTOR and FoxO signaling pathways were significantly enriched (Fig. 3C). Given the crosstalk among these three pathways, an analysis of overlapped genes was conducted and 16 genes were identified, including SGK1 and PI3K (Pik3cb, Pik3ca, Pik3r1) (Fig. 3D), Previous studies have shown that SGK1 is a downstream effector of PI3K [25–28]. As a consequence, we speculated that PI3K-SGK1 might be responsible for Dex-induced metastasis. Dex treatment increased the activation level of PI3K in vitro (Fig. 3E), and wortmanin, the PI3K inhibitor, reduced Dex-induced cell migration (Fig. 3F) without affecting the proliferation of 4T1 cells (Supplementary Fig. S4), indicating that Dex promoted cell migration through the PI3K pathway. Meanwhile, consistent result was observed using another PI3K inhibitor GDC0941 (Supplementary Fig. S5). A previous study has reported that Dex promotes breast cancer metastasis via the Wnt pathway and its downstream gene, tyrosine-protein kinase transmembrane receptor *ROR1*, which was also upregulated in our study [15]. However, PI3K inhibition did not change *ROR1* expression (Fig. 3G). These results suggested that the PI3K pathway may be a new and independent mechanism in Dex-induced metastasis.

Based on the results presented in Supplementary Figs. S3 and 2E, it was found that Dex has little effect on EMT and invasion by breast cancer cells. Therefore, we speculate that Dex may promote tumor metastasis by affecting tumor cell adhesion. Studies have suggested that tumor cell adhesion is a prerequisite for tumor metastasis. Here, 17 genes, including *CTGF* and integrin-related genes, were identified according to overlap involving cell adhesion, cell-matrix adhesin, and cell migration (Fig. 3H). We selected *CTGF* as a potential target because it is associated with cell differentiation, proliferation, and metastasis [29–31] (Fig. 3I).

Hence, these results indicated a new mechanism of Dex-induced metastasis, which might rely on the PI3K pathway and might involve *SGK1* and *CTGF*.

### SGK1 mediated breast cancer metastasis

**Fig. 4:**
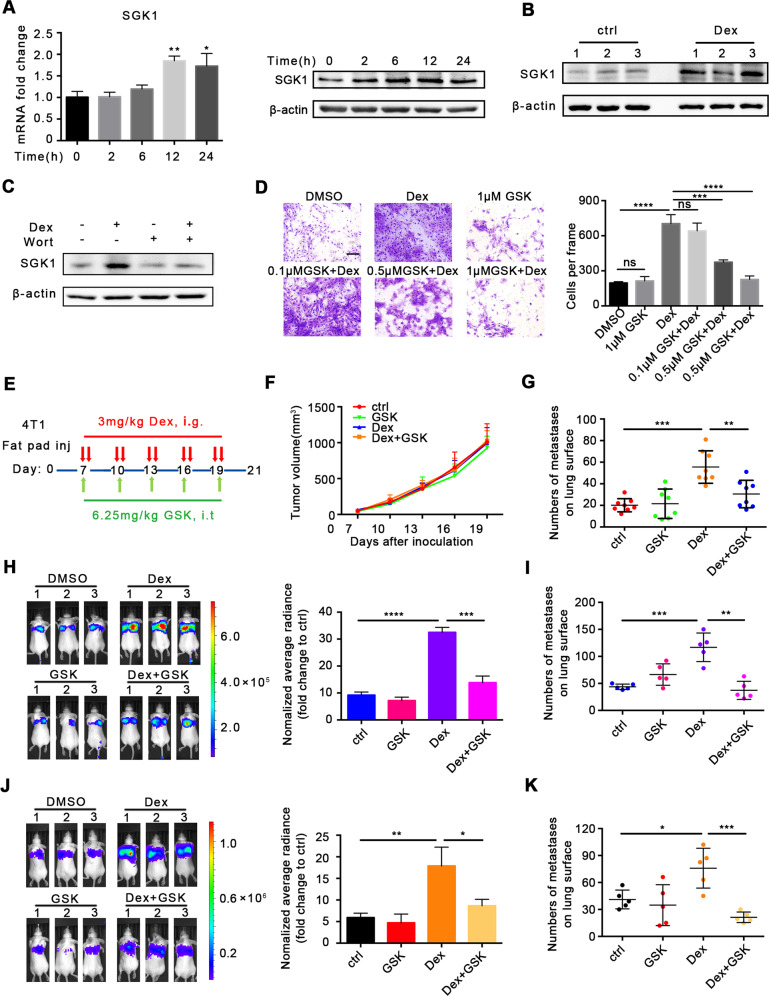
SGK1 mediates Dex-induced breast cancer metastasis. **A** The expression of SGK1 in 4T1 cells after Dex treatment. **B** The expression of SGK1 in 4T1 tumor tissue. **C** The SGK1 expression after Dex/Wortmannin treatment. **D** The influence of SGK1 inhibitor GSK650394 on the migration of 4T1 cells. The scale bar is 25 μm, *n* = 3 replicates. **E** Treatment scheme. **F** Tumor growth. (*n* = 8 mice per group). **G** The number of metastases on lung surface (*n* = 8 mice per group). **H**, **I** The effect of GSK650394 on experimental lung metastasis model. **H** Representative image of bioluminescence and statistics were shown (*n* = 3 mice per group). **I** The number of metastases on lung surface (*n* = 5 mice per group). **J**, **K** 4T1 cells were treated with Dex/ GSK650394 in vitro for 48 h before inoculation via intravenous injection. **J** Representative image of bioluminescence and statistics were shown (*n* = 3 mice per group). **K** The number of metastases on lung surface (*n* = 5 mice per group). Mean ± SEM is shown. *p < 0.05, **p < 0.01, ***p < 0.001, ****p < 0.0001.

Based on the above findings, we first determined *SGK1* expression in cells with Dex treatment. Both mRNA and protein levels of SGK1 were upregulated in mouse and human breast cancer cell lines treated with Dex (Fig. 4A, Supplementary Fig. S6A). Moreover, SGK1 expression in 4T1 orthotopic tumor tissue was also augmented after Dex treatment (Fig. 4B). To clarify whether *SGK1* was regulated by PI3K, which was indicated by sequence analysis, we inhibited PI3K with wortmanin [32], and found that wortmanin suppressed the increased expression of *SGK1* caused by Dex treatment (Fig. 4C), suggesting that Dex upregulated the expression of *SGK1* via PI3K signaling.

We then explored the effects of SGK1 on Dex-induced metastasis. GSK650394, a SGK1 inhibitor, significantly inhibited *SGK1* expression and Dex-induced cell migration (Supplementary Fig. S7B and Fig. 4D) but with no effects on cell proliferation (Supplementary Fig. S7A). Similar results were also observed with human breast cancer cells (Supplementary Fig. S8A, B). It was found that in 4T1 cells with *SGK1* knockout using CRISPR/Cas9 could effectively inhibited the enhancement of cell adhesion and migration caused by Dex in 4T1 cells (Supplementary Fig. S9A, B). To further study the effects of SGK1 in vivo, we established a spontaneous lung metastasis model in 4T1 cells in situ, and treated mice with GSK650394 following a defined schedule (Fig. 4E). The results showed that, although GSK650394 could inhibit SGK1 expression in vivo (Supplementary Fig. S7C, D), it did not affect tumor growth (Fig. 4F) or weight (Supplementary Fig. S10A), whereas the number of metastases on the lung surface in the Dex+GSK group was significantly decreased compared with that in the Dex-only group (Fig. 4G and Supplementary Fig. S10B, C). Furthermore, in an experimental metastasis model (Supplementary Fig. S11A), SGK1 inhibition remarkably suppressed Dex-induced metastasis (Fig. 4H, I and Supplementary Fig. S11B). As shown in Fig. 2D, once the breast cancer cells were primed, Dex maintenance was not necessary for cell migration. In order to test whether this effect was present in vivo, we treated mice following the scheme (Supplementary Fig. S11C). As expected, 4T1 cells after Dex pre-treatment exhibited more metastatic signals and nodes in the experimental metastasis model, and pre-treatment with GSK650394 in vitro attenuated these effects (Fig. 4J, K, Supplementary Fig. S11D).

Taken together, these data demonstrated that SGK1 was required for Dex-induced metastasis.

### Dex promotes metastasis through upregulation of CTGF expression

**Fig. 5:**
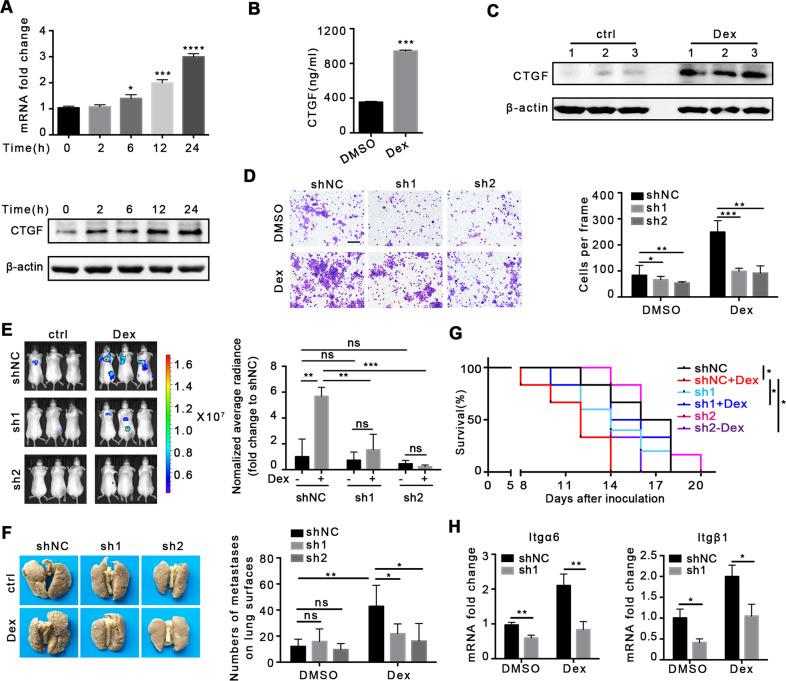
Dex promotes metastasis through upregulation of CTGF expression. **A** The expression of CTGF in 4T1 cells after Dex treatment. **B** The concentration of CTGF in 4T1 cell supernatant after Dex treatment (24 h). **C** The expression of CTGF in 4T1 tumor tissue. **D** The effect of CTGF knockdown using shRNA on 4T1 cell migration, *n* = 3 replicates, the scale bar is 25 μm. **E** Representative image of bioluminescence and average radiance in experimental lung metastasis (*n* = 3 mice per group). **F** Typical mouse lung tissue fixed with Bouin’s fixation and the number of metastases on lung surface (*n* = 5 mice per group). **G** Survival (*n* = 6 mice per group). **H** The expression of Itgα6 and Itgβ1 in 4T1 cells in vitro, *n* = 3 replicates. Mean ± SEM is shown. *p < 0.05, **p < 0.01, ***p < 0.001; ns no statistical significance.

The cell adhesion molecules including selectin, cadherin, and integrin are closely involved in cancer metastasis [33–36]. As shown in Fig. 3H, I, we identified CTGF as a potential target in Dex-induced metastasis. To verify this, we first determined CTGF expression after Dex treatment. It was found that Dex treatment upregulated both CTGF mRNA and protein levels in 4T1 cells (Fig. 5A), and an increased concentration of secretory CTGF was observed in the supernatant (Fig. 5B). In addition, enhanced CTGF after Dex treatment was observed in MDA-MB-231 human breast cells (Supplementary Fig. S6B). Detection of CTGF in tumor tissue in the 4T1 orthotopic model after Dex treatment also showed similar results (Fig. 5C). In order to further investigate the role of CTGF in metastasis, we successfully designed two shRNAs targeting *CTGF* (Supplementary Fig. S12A, B). Results showed that *CTGF* knockdown inhibited Dex-induced cell migration (Fig. 5D). In an experimental metastasis model, 4T1 cells were pre-treated with 100 nM Dex for 48 h, and then used for intravenous injection. It was found that *CTGF* knockdown significantly reduced pulmonary tumor signals and metastatic nodes (Fig. 5E, F) and prolonged mouse survival (Fig. 5G and Supplementary Fig. S13A–E). The above results indicated that Dex promoted the metastasis of 4T1 cells through CTGF.

CTGF can act as an integrin ligand to mediate downstream signals that regulate cytoskeletal and cell migration functions [37–39]. Data presented in Fig. 3I suggest an increased expression of integrin subunits after Dex treatment. To test whether CTGF mediated the expression of integrin, we detected mRNA expression of integrin subunit transcripts. The results indicated that Dex treatment upregulated the expression of integrins Itgα6 and Itgβ1, and CTGF knockdown downregulated these genes (Fig. 5H).

Therefore, these data suggested that upregulated CTGF expression and downstream integrin expression are responsible for Dex-induced metastasis.

### SGK1 regulates CTGF expression

**Fig. 6:**
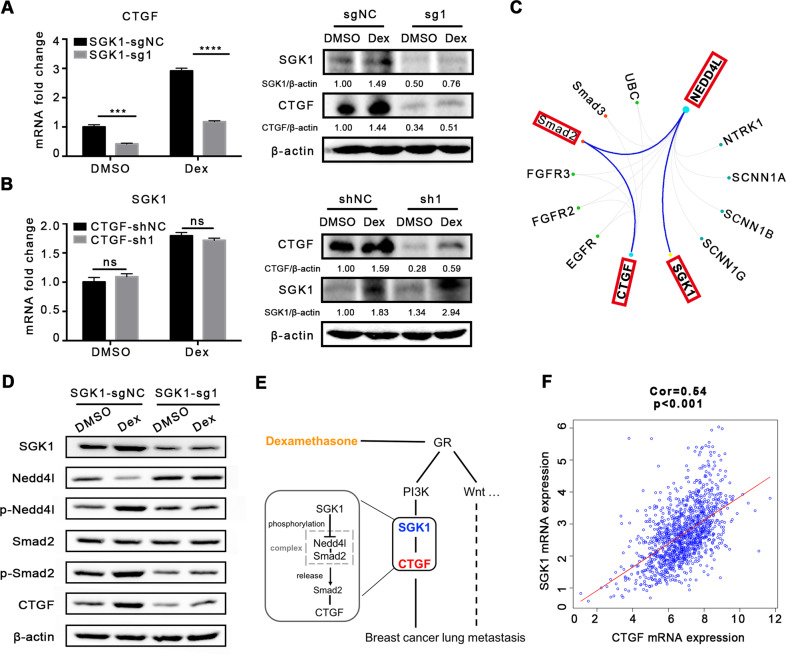
SGK1 regulates CTGF expression. **A** The expression of CTGF after SGK1 knockout using CRISPR/Cas9, *n* = 3 replicates. **B** The expression of SGK1 after CTGF knockdown using shRNA, *n* = 3 replicates. **C** Schematic diagram of the molecular regulatory network between SGK1 and CTGF. **D** The expression of Nedd4l, p-Nedd4l, Smad2 and p-Smad2 after SGK1 knockout. **E** The mechanism of Dex-induced lung metastasis. **F** The correlation between SGK1 and CTGF expression in breast cancer. Mean ± SEM is shown. ***p < 0.001, ****p < 0.0001; ns no statistical significance.

Based on the fact that both SGK1 and CTGF were involved in the process of Dex-induced breast cancer metastasis, we queried whether crosstalk exists between these two key genes. First, we detected CTGF expression in 4T1 cells with SGK1 knockout using CRISPR/Cas9. It was found that SGK1 knockout downregulated CTGF expression at both the mRNA and protein levels (Fig. 6A), and that *CTGF* knockdown did not affect SGK1 expression (Fig. 6B), suggesting that CTGF expression was regulated by SGK1.

To investigate mechanisms linking signals between SGK1 and CTGF, analysis of multigene radar function in GCBI indicated that Neddd4l (Nedd4 Like E3 Ubiquitin Protein Ligase) and Smad2/3 might play roles in regulating CTGF expression by SGK1 (Fig. 6C). Moreover, previous studies have reported that, in HEK293T cells, Neddd4l binds to Smad2/3 to form a complex and subsequently inhibits Smad2/3 signaling and expression of its downstream genes (*Smad7, Pai-1*, and *CTGF*) [40]. While SGK1 can bind to, and reduce the accumulation of, Nedd4l through phosphorylation [41, 42], this evidence suggested that SGK1 might regulate CTGF through Nedd4l and Smad2 in Dex-induced breast cancer metastasis. To verify this hypothesis, 4T1 cells with SGK1 knockout were used for analysis, as shown in Fig. 6D. Total Nedd4l was reduced after Dex treatment, which was accompanied by increased p-Nedd4l levels, in addition, Dex treatment increased p-Smad2 levels, and *SGK1* knockout abolished these changes, suggesting that CTGF was regulated by SGK1.

Collectively, SGK1 expression was upregulated by the PI3K pathway activated by ligation of Dex and GR, and then SGK1 phosphorylated Nedd4l, which led to Smad2 release from the suppressive Nedd4l-Smad2 complex, and subsequent upregulation of CTGF, which finally promoted tumor metastasis (Fig. 6E). In addition, analysis using the GSEA (Gene Set Enrichment Analysis) database revealed that the expression of SGK1 and CTGF was positively correlated in breast cancer (*P* < 0.001) (Fig. 6F).

### Dex is a major factor in breast cancer metastasis in standard PTX chemotherapy

**Fig. 7:**
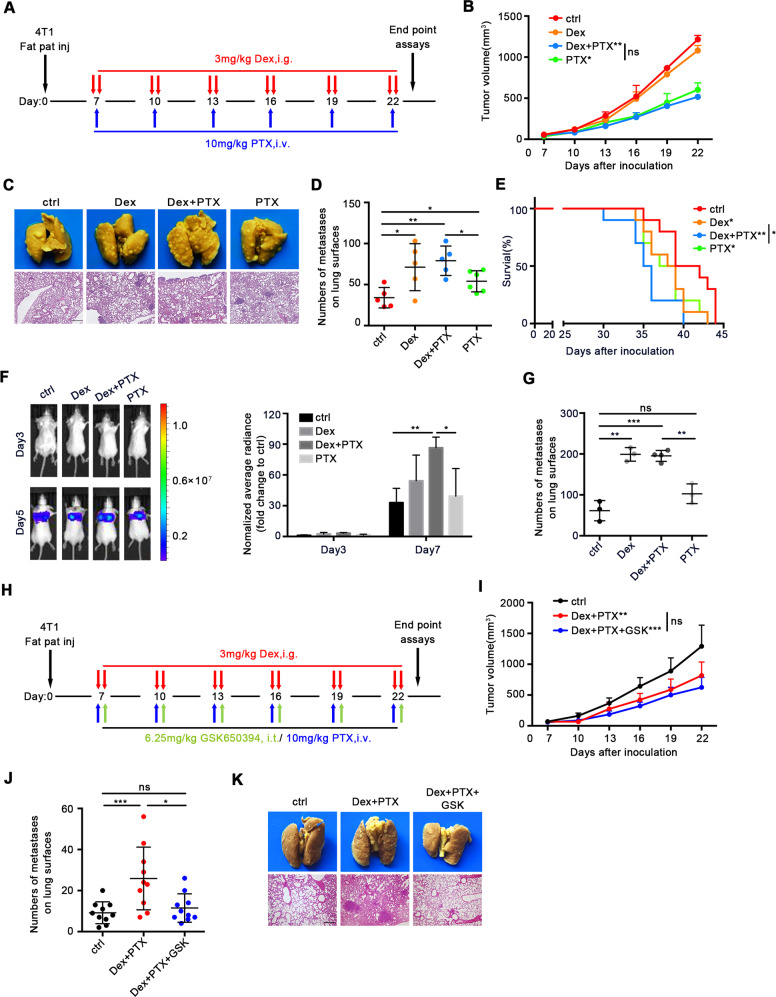
SGK1 is a potential therapeutic target for PTX chemotherapy-induced metastasis. **A** Schematic diagram of Dex+PTX. **B** Tumor volume, *n* = 5 mice per group. **C**–**E** The role of Dex in the treatment of breast cancer with Dex+PTX. **C** Typical mouse lung tissue fixed with Bouin’s fixation and H&E staining of lung. **D** The number of metastases on lung surface (*n* = 5 mice per group). **E** Survival (*n* = 10 mice per group). **F** Representative image of bioluminescence and statistics were shown (*n* = 3 mice per group). **G** The number of metastases on lung surface (*n* = 5 mice per group). **H** Schematic diagram of Dex+PTX+GSK650394. **I**–**K** The effect of GSK650394 in the treatment of breast cancer with Dex+PTX. **I** Tumor volume (*n* = 10 mice per group). **J** Typical mouse lung tissue fixed with Bouin’s fixation and the H&E staining of lung (*n* = 10 mice per group). **K** The number of metastases on lung surface (*n* = 10 mice per group). Mean ± SEM is shown. *p < 0.05, **p < 0.01, ***p < 0.001, ****p < 0.0001; ns no statistical significance.

Hypersensitivity reaction to paclitaxel (PTX) is frequently encountered in cancer patients, and Dex has been shown to reduce significantly the risk [43]. Our present data has proven the key role of Dex in breast cancer metastasis, and we tried to investigate the effect of Dex in chemotherapy regimen containing PTX. Orthotopic 4T1-bearing mice were treated using regimen similar with clinical practice (Fig. 7A), which Dex (3 mg/kg) was administered before PTX injection. It was found that Dex did not influence the antitumor effects of PTX (Fig. 7B). However, as shown in Fig. 7C, D, compared with PTX treatment alone, mice receiving Dex+PTX treatment showed greater numbers of metastatic nodes. PTX treatment also increased breast cancer metastasis, which was consistent with findings of previous studies [4, 5]. Notably, the absence of variation between Dex and Dex+PTX treatments demonstrated that Dex played a dominant role in breast cancer metastasis caused by standard PTX chemotherapy. Furthermore, despite effective tumor control by PTX chemotherapy, mice treated with Dex+PTX exhibited the shortest overall survival compared with the other groups (Fig. 7E). In an experimental metastasis model, the Dex+PTX group also exhibited more abundant tumor signals and nodes in the lung when compared with PTX treatment alone (Fig. 7F, G).

Hence, our results showed that Dex was a leading factor that promoted breast cancer metastasis in the standard PTX regimen, which should raise concerns in clinical breast cancer treatment.

### SGK1 is a potential therapeutic target for PTX chemotherapy-induced metastasis

Based on the data in Fig. 4G that SGK1 inhibition could block Dex-induced metastasis, we evaluated the potential therapeutic effects of SGK1 inhibitor GSK650394 in Dex+PTX chemotherapy. Mice were treated following the scheme (Fig. 7H), and the results showed that GSK650394 did not affect the antitumor effects of Dex+PTX (Fig. 7I), but, remarkably, inhibited lung metastases caused by Dex+PTX chemotherapy (Fig. 7J, K). These data indicated that SGK1 could be used as a potential target to treat Dex+PTX chemotherapy-induced breast cancer metastasis.

## Discussion

Metastasis severely affects the lives and health of breast cancer patients. Triple-negative breast cancer (ER-/PR-/HER2-) accounts for 10–15% of the clinical incidence of breast cancer with high malignancy and poor prognosis [44]. Single cytotoxic agent chemotherapy remains the standard regimen for patients with metastatic triple-negative breast cancer [45–47]. However, recent studies indicated that some breast cancer patients underwent metastasis after receiving treatment, and paclitaxel played a “double-edged sword” role, which conferred both cytotoxic and pro-metastatic effects in cancer cells [4, 5]. However, most concerns surrounding metastasis are focussed on the drug itself or genetic mechanisms, and the role of adjuvant drugs is less well known. In the present study, we provided evidence that Dex, an adjuvant drug widely used to reduce adverse reactions to chemotherapy, could promote breast cancer lung metastasis via the PI3K-SGK1-CTGF pathway. Of note, our data also proved that Dex showed greater ability to promote metastasis than did PTX in standard PTX chemotherapy, which was indicated by more metastatic nodes and shorter overall survival in mice treated with Dex+PTX.

Cancer metastasis is an intricate process involved in both the cancer cell intrinsic pathway and extrinsic cellular factors, including immune cells and cytokines [48–50]. Our results suggested that the pro-metastatic effect of Dex is independent of tumor-associated immune cells, which is closely linked to cancer metastasis. Recently, atezolizumab and nab-paclitaxel combination therapy was reported to be a promising regimen in previously untreated patients with metastatic triple-negative breast cancer [45]. Our results indicated that the appropriate dose of Dex had little impact in immune cells and thus might not affect the efficacy of immunotherapy. Moreover, EMT has been reported as a key mechanism involved in metastasis [51]. In recent years, the role of EMT in breast cancer metastasis has been controversial. Fisher et al. found that lung metastases were composed of non-EMT tumor cells in a model of epithelial primary breast cancer by establishing a tracking system using interstitial cell-specific Cre-mediated fluorescence [52]. In our study, we found that Dex treatment did not alter the EMT process. Dex promoted the migration of breast cancer cells rather than invasion, indicating that degradation of the extracellular matrix was not affected by Dex. Further evidence suggested that Dex promoted breast cancer metastasis via CTGF-mediated cell adhesion and colonization in the lung rather than the process of EMT.

Dex is a long-lasting and cost-effective glucocorticoid and has been widely used as an adjuvant or therapeutic agent in clinical settings. In hematological malignancies, Dex alone or in combination with chemotherapy shows effective therapeutic outcomes. In addition, Dex can effectively inhibit the growth of colorectal cancer xenografts in nude mice by increasing the retention time of drugs in the tumor [53, 54]. Some studies have reported that Dex can increase the resistance of breast cancer cells to chemotherapy and promote tumor cell survival in hostile environments [55, 56]. We also found that Dex could suppress apoptosis of breast cancer cells in response to low glucose and chemotherapeutic drugs (data not shown). To date, little is known regarding the links between Dex and breast cancer metastasis. A recent study reported that the GC content in serum of breast cancer patients with metastasis is significantly higher than in non-metastatic breast cancer patients, with increased ROR1 expression and Wnt pathway activation induced by GR activation-mediated breast cancer metastasis [15], which was also found from our transcriptome sequencing results. In this study, we revealed a new and independent mechanism in Dex-induced metastasis, in which Dex activated the PI3K pathway through GR and then upregulated SGK1 expression, increased SGK1 levels and enhanced CTGF expression through Nedd4l-Smad2. PI3K inhibition did not interfere with ROR1 expression. Moreover, in a Src kinase -omics identification analysis of MCF10A breast cancer epithelial cells, it was found that ROR1 and SGK1 are located in different regulatory branches [57], thus suggesting an independent pathway as revealed by our study.

Dex pre-treatment before PTX administration is a standard procedure in breast cancer treatment, performed with the intent of reducing allergic responses, nausea, and vomiting caused by paclitaxel. Chemotherapeutic drug-induced metastasis has been reported previously. However, data from our study showed that Dex conferred stronger prometastatic ability than did drugs in standard PTX chemotherapy. These results should raise marked concerns in clinical use of PTX or other chemotherapy in breast cancer patients. Based on these findings, it can be concluded that modification of PTX formulations to reduce or abolish Dex application is the optimal choice. Recently, many efforts have been exerted in this field to attenuate the side effects of chemotherapeutic drugs. For example, preparations involving nanotechnology and albumin is a promising strategy and has shown benefits in clinical and preclinical models. Nevertheless, Dex is still a widely used pre-treatment agent in chemotherapy with regard to its long-lasting effects and low cost for most patients. Currently, Dex is still a commonly used pre-treatment agent in tumor therapy, which effectively reduces the symptoms of nervous system compression and chemotherapy-related vomiting, and prevents radiotherapy-related toxicity and other side effects. In this study, we found that targeting SGK1 with GSK650394 could inhibit Dex-induced lung metastasis without affecting antitumor capacity, although intratumoral injection of GSK650394 in our study was not an optimal choice. Our results have strengthened the concept that SGK1 might be a potential candidate to overcome chemotherapy-induced metastasis in breast cancer. Efforts to explore new formulations of chemotherapeutic agents to reduce side effects and develop potential therapeutic targets are of considerable significance for breast cancer treatment. In addition, previous studies also suggested SGK1 was important for breast cancer growth and resistance to PI3K inhibitors [27, 28], indicating that targeting SGK1 would generate pleiotropic antitumor effects.

Our study uncovered a new mechanism by which Dex exacerbates lung metastasis by breast cancer cells through PI3K-SGK1-CTGF signaling and raises concerns about the risk of Dex pre-treatment in breast cancer chemotherapy. Meanwhile, our present data also provides strategy targeting SGK1 to suppress Dex-induced lung metastasis. Considering corticosteroids such as Dex are widely used in various cancers to treat the side effects of chemotherapy and to combat symptoms related to advanced cancer. Thus, assessment of the side effects of hormone pathways on metastasis is also warranted for other types of cancer.

## Materials and methods

### Mice and cell lines

6- to 8-week-old female BALB/c mice and BALB/c nude mice were purchased from Beijing Huafukang Bioscience (Beijing, China). The mouse breast cancer cell line 4T1, human breast cancer lines MCF-7, MDA-MB-231, and ZR-75-30 were purchased from American Type Culture Collection (ATCC). 4T1-luc and MDA-MB-231-luc were generated using lentivirus expressing luciferase, purchased from Hanbio (Shanghai, China). 4T1 cell with CTGF knockdown or SGK1 knockout was constructed by shRNA and CRISPR/Cas9 respectively, which m-CTGF-shRNA was purchased from Hanbio (Shanghai, China) and m-AVC-LW983-sgSGK1/NC was purchased from Syngentech (Beijing, China). Information for primer sequences is provided in Supplementary Table 1.

### Tumor model and treatment

A total of 2 × 10^5^ 4T1 cells in 60 μl serum-free medium were inoculated into the second mammary fat pad on the right side of BALB/c mice or BALB/c nude mice to establish a spontaneous lung metastasis model. Tumor sizes were measured using a digital calliper every three days, and mice were terminated when tumor volumes reached 1500 mm^3^. Mice that succumbed to tumor were included in calculations of overall survival.

For the experimental metastasis model, 2 × 10^5^ 4T1-luc cells/ and 1 × 10^6^ MDA-MB-231-luc cells in 200 μl serum-free medium were injected intravenously (i.v.) into BABL/c mice and BABL/c nude mice, respectively. Tumor development was monitored using IVIS Lumina III (Perkin Elmer, Waltham Mass, USA) after intraperitoneal (i.p.) injection of d-Luciferin (Gold Biotechnology, Missouri, USA).

For in situ treatment in the 4T1 tumor model, Dex (MedChemExpress, New Jersey, USA) treatment (3 mg/kg) was performed every three days by gavage twice/day when the tumors reached 50–100 mm^3^; paclitaxel (i.v. 10 mg/kg) was injected 6 h after the latest Dex administration, while delivery of GSK650394 (MedChemExpress, New Jersey, USA) (i.p. 6.25 mg/kg) was performed on the same day. The experimental metastasis model was administered, as described previously. All experiments were performed in accordance with the guidelines of the Animal Care and Use Committee of West China Hospital, Sichuan University, China.

### Migration and invasion assays

For vertical migration assays, 5 × 10^4^ cells were suspended in 100 μl serum-free DMEM medium and seeded into the upper chamber of each Transwell (8.0 μm) insert, and 700 μl of DMEM containing 10% FBS was added to the lower chamber in 24-well plates. After incubation at 37 °C for 48 h, cells that had migrated were fixed with 4% paraformaldehyde and stained with 0.1% (w/v) Crystal Violet for 15 min.

For invasion assays, chambers were uniformly covered with 60 μl Matrigel diluted with DMEM (1:8) and incubated at 37 °C for 2 h. Then, 5 × 10^4^ cells were suspended in 100 μl DMEM and seeded in the upper chambers, and 700 μl of DMEM containing 10% FBS was added to the lower chambers. After incubation at 37 °C, the cells were fixed and stained.

### Western blotting

Proteins were extracted from cells with treatment as indicated using RIPA lysis buffer (Beyotime, Nanjing, China) containing 1% protease inhibitor cocktail (Merck Millipore, Birrika, USA), and then prepared for sample loading with buffer. After SDS- PAGE, proteins were transferred onto polyvinylidene difluoride membranes (Merck Millipore). The antibodies used for western blotting are listed in Supplementary Table 2. All antibodies were used at a 1:1,000 dilution. β-actin was used as a loading control for all experiments, with an HRP-linked secondary antibody (ZSGB-BIO, Beijing, China).

### Real-time PCR

Total RNA was extracted using TRIzol (Life Technologies, Massachusettes, USA). After measuring the concentration, RNA was reverse transcribed, and mRNA expression analysis was performed using PrimeScript RT Reagent Kit (TaKaRa, Japan) on a LightCycler 96 System (Roche, Basel, Swizerland) with the following PCR conditions: 95 °C for 150 s; 40 cycles of 95 °C for 10 s, 58 °C for 30 s, 95 °C for 10 s, 65 °C for 60 s, and 97 °C for 1 s. Gene expression was normalized to the housekeeping gene β-actin. Both forward and reverse primers are listed in Supplementary Table 3.

### Statistical analysis

The data were analyzed using Prism (GraphPad Prism version 5). Statistical significance was analyzed using unpaired Student’s *t* test. Animal survival was presented using Kaplan–Meier survival curves and analyzed using the log-rank test. A value of *p* < 0.05 was considered statistically significant. In the figures, the symbols used to denote significance are as follows: *p < 0.05, **p < 0.01, ***p < 0.001, ****p < 0.0001, and ns (no statistical significance).

## Supplementary information

Supplemental materials and figures
