# Supplementary material for: Dexamethasone enhances the lung metastasis of breast cancer via a PI3K-SGK1-CTGF pathway
Source: Oncogene. 2021 Jul 16;40(35):5367–78. doi: 10.1038/s41388-021-01944-w (PMC8413128; doi:10.1038/s41388-021-01944-w)
Supplement: Supplementary file 1 — Supplemental materials and figures [file 41388_2021_1944_MOESM1_ESM.pdf]

Supplementary materials and methods

Cell proliferation assays

Cells were seeded at a density of 2000 cells/ well in 96-well plates and incubated at 37°C. Then Cell Counting Kit-8 (Dojindo, Shanghai, China) reagents were added to the wells and incubated for 3 h. Optical absorbance was measured at 450 nm to calculate the number of viable cells in each well. Each measurement was performed in triplicate and the experiments were repeated three times.

Colony formation assays

Cells were seeded in six-well plates at a density of 500 cells/ well and cultured at 37°C for two weeks. At the end of incubation, the cells were fixed with 4% paraformaldehyde and stained with 0.1% (w/v) crystal violet. Each measurement was performed in triplicate and the experiments were repeated three times.

Immunohistochemical (IHC) staining

Tumor and lung tissues were fixed in 4% paraformaldehyde and Bouin's fixative at room temperature for 48 h, dehydrated across an alcohol gradient, and embedded with a Leica tissue embedder. Four-millimetre thick sections were cut and transferred to a tissue flotation bath. Slides were then dried at room temperature overnight and stored at 4°C. Slides were incubated at 60°C for 10 min to melt the paraffin and subjected to deparaffinization (xylene, 10 min, 2 times; 100% ethanol, 3 min; 95% ethanol, 3 min; 85% ethanol, 3 min; 75% ethanol, 3 min; and water, 3 min). The slides were then incubated with H_2O_2 to quench endogenous peroxidase activity. Heat-mediated antigen unmasking was performed in citrate buffer. After blocking with goat serum, the sections were incubated with primary antibodies against SGK1 (1:100, Huabio) in a moisture chamber overnight at 4°C. The next day, the slides were stained with horseradish peroxidase (HRP)-labelled secondary antibody for 2 h. We visualised staining using 3,3'-diaminobenzidine (DAB), and positive cells were indicated by the presence of brown staining in both the nucleus and cytoplasm, while the nucleus was stained with haematoxylin.

Enzyme-linked immunosorbent assays

Supernatants from the cancer cells were collected, and cell numbers were counted

using a Countstar instrument (Ruiyu, Shanghai, China). The supernatant was used to measure the total levels of several cytokines using a mouse CTGF ELISA kit (Elabscience, Wuhan, China), according to the manufacturer's instructions. Cytokine expression levels (pg/ml) per 5 $\times 10^5$ cancer cells were analyzed.

Cell adhesion assays

Cells were seeded at a density of 10^4 cells/ well in 96-well plates and incubated at 37°C. When the cells begin to adhere under the microscope (about 30-60 minutes), washed gently with pre-warmed PBS to remove the cells that weakly adherent. Add CCK8 reagent to each well and incubated at 37°C. Optical absorbance was measured at 450 nm 3 hours later.

Supplementary Figures

Figure S1

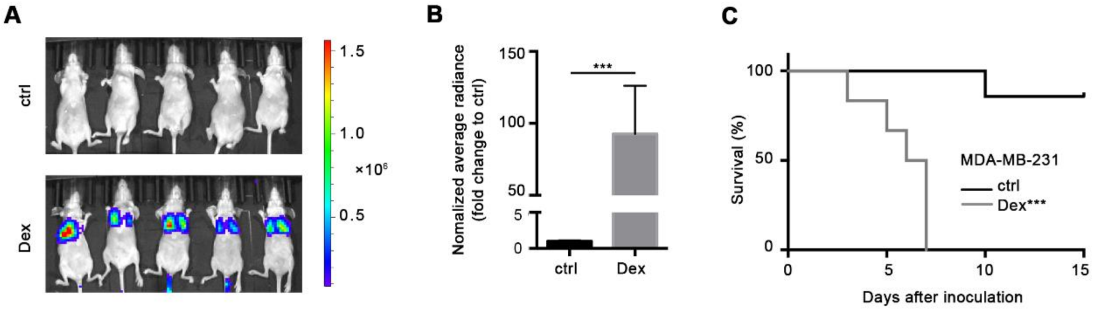

Figure S1. Dex increases metastatic colonization and reduces survival.

(A-B) The effect of Dex-induced lung colonization upon intravenous injection with MDA-MB-231-luc was evaluated by in vivo bioluminescence on the day of 7. (A) Representative image of bioluminescence and (B) average radiance, n=5 mice per group. (C) Survival, n=5 mice per group. Mean ± SEM is shown. ***p < 0.001.

Figure S2

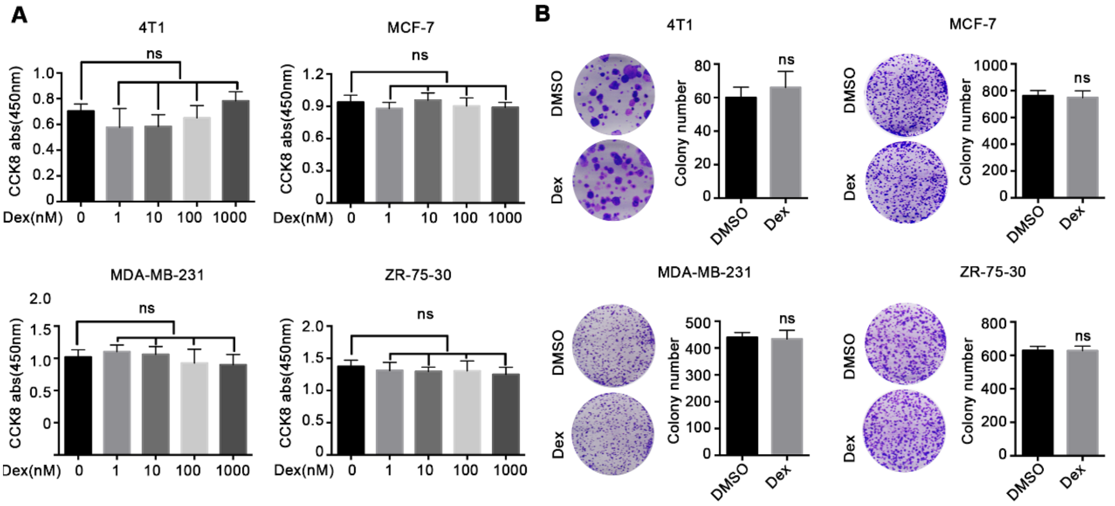

Figure S2. Dex has little effect on the proliferation and colony forming ability of breast cancer cells without additional estradiol. (A) The effect of Dex on the proliferation of 4T1, MCF-7, MDA-MB-231 and ZR-75-30. Cells were seeded at a density of 2000 cells/ well in 96-well plates and incubated at 37°C for 48h. The change of cell proliferation was observed with CCK8 colorimeter. n=3 replicates. (B) The effect of Dex on the colony forming ability of 4T1, MCF-7, MDA-MB-231 and ZR-75-30. n=3 replicates. Mean ± SEM is shown, ns, no statistical significance.

Figure S3

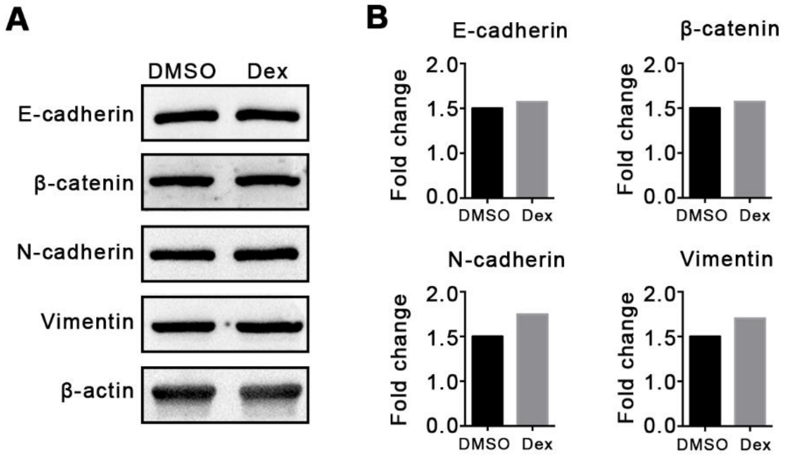

Figure S3. Dex does not induce EMT in 4T1 cells. (A) The 4T1 cells were treated with 100nM Dex for 48 hours and expression of EMT-related markers including E-cadherin, β-catanin, N-cadherin and Vimentin were tested by Western Blot. (B) Grayscale statistics.

Figure S4

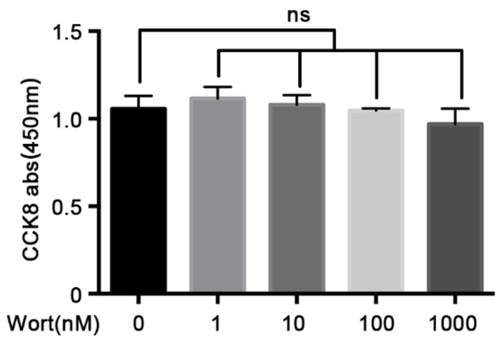

Figure S4. The effects of Wortmannin on cell proliferation. Cells were seeded at a density of 2000 cells/ well in 96-well plates and incubated at 37°C for 48h. The change of cell proliferation was observed with CCK8 colorimeter. n=3 replicates. Mean ± SEM is shown, ns, no statistical significance.

Figure S5

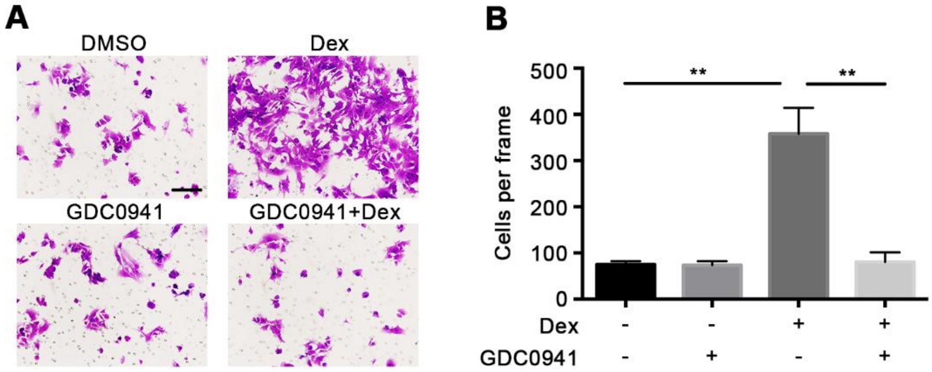

Figure S5. The effects of GDC0941 on Dex-induced cell migration. (A) 4T1 cells were treated with 100nM Dex or GDC0941(100 nM) for 48 hours. The scale bar is 25μm, n=3 replicates. (B) Statistics. Mean ± SEM is shown. **p < 0.01

Figure S6

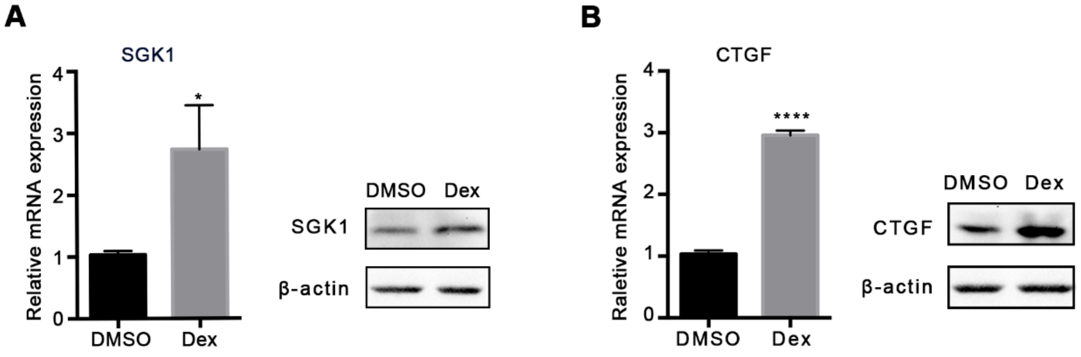

Figure S6. Dex increases the expression of SGK1 and CTGF in human breast cancer MDA-MB-231. The expression of SGK1(A) and CTGF(B) in MDA-MB-231 cells after Dex treatment. Mean ± SEM is shown. *p < 0.05, ****p < 0.0001.

Figure S7

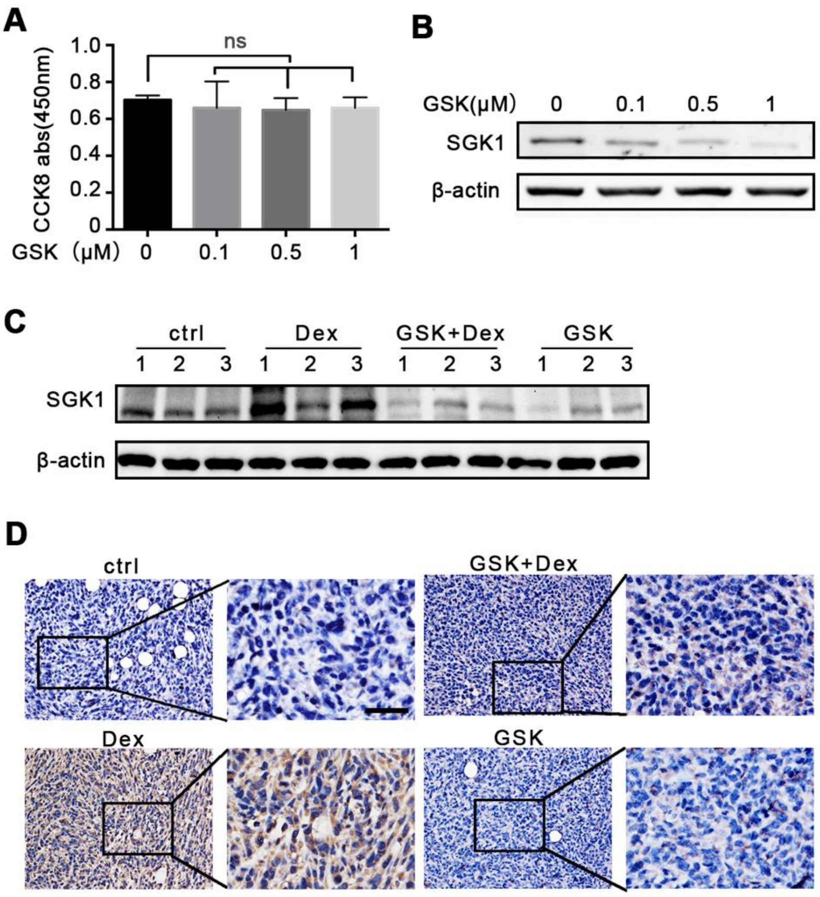

Figure S7. The effect of GSK650394 *in vitro* and *in vivo*. (A) Cell proliferation, n=3 replicates. (B) The expression of SGK1with different concentrations of GSK650394 treatment. (C) Western blot detection of SGK1 expression in 4T1 tumor tissue. (D) IHC

detection of SGK1 expression in 4T1 tumor tissue, the scale bar is 100μm. The scale is 100μm. Mean ± SEM is shown, ns, no statistical significance.

Figure S8

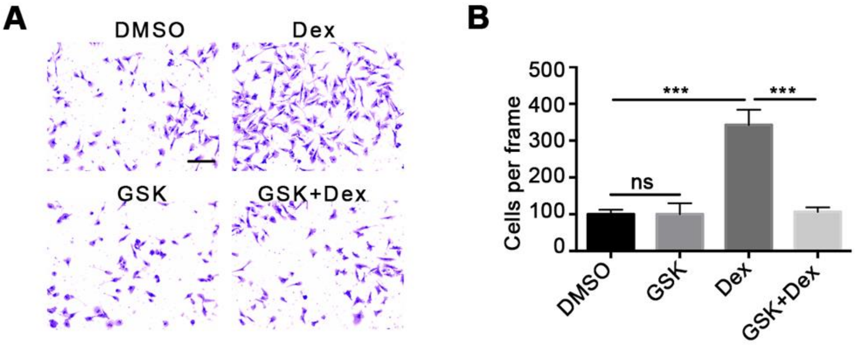

Figure S8. The effect of GSK650394 on the migration of MDA-MB-231 cells with Dex treatment. (A) Cell migration. The scale is 25μm. (B) Statistics. Mean ± SEM is shown. ***p < 0.001; ns, no statistical significance.

Figure S9

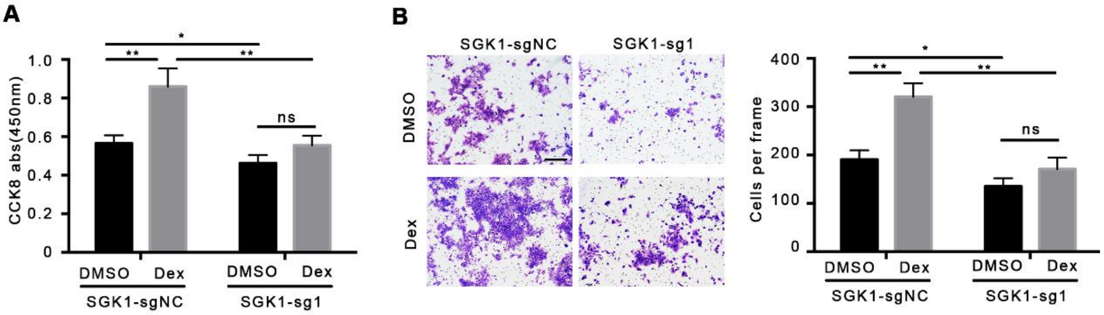

Figure S9. SGK1 knockout inhibits Dex-induced 4T1 cell migration. (A) Cell adhesion. (B) Cell migration and statistics. Mean ± SEM is shown. *p < 0.05, **p < 0.01; ns, no statistical significance.

Figure S10

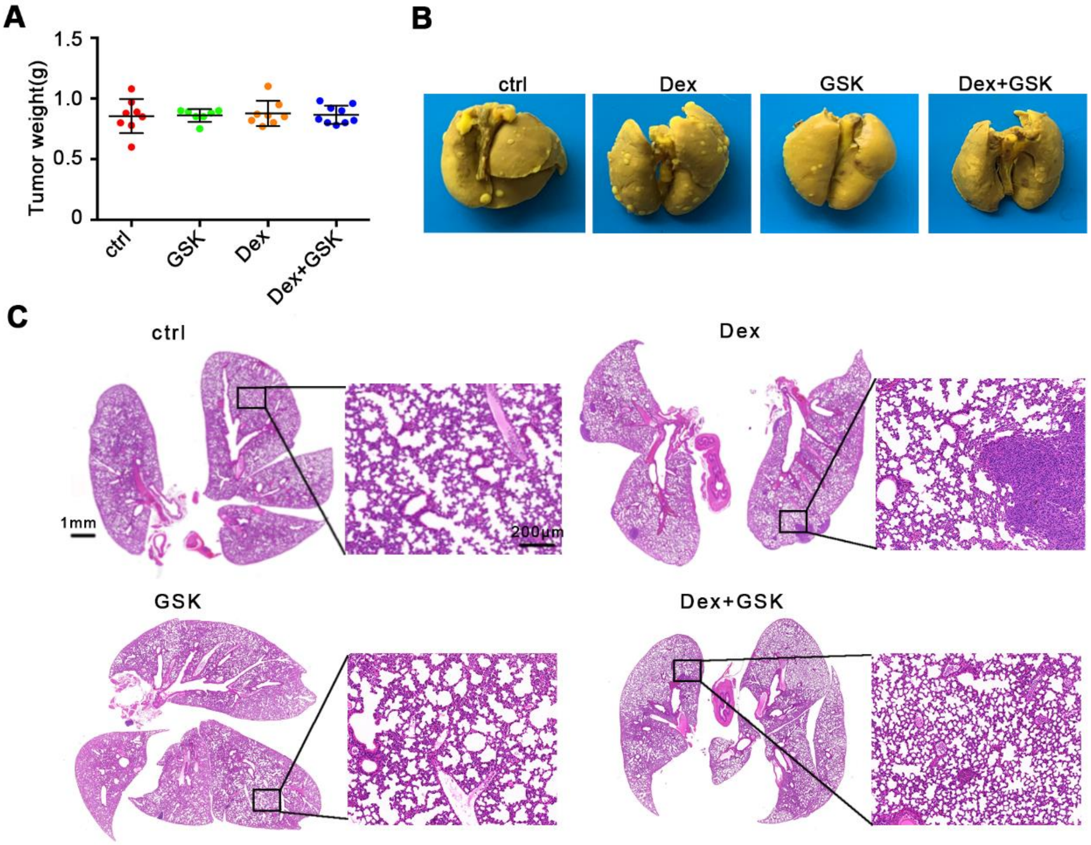

Figure S10. GSK650394 inhibits lung metastasis in orthotopic model. (A) Tumor weight, n=8 mice for each group. (B) Typical mouse lung tissue fixed with Bouin's fixation. (C) H&E staining of lung tissue.

Figure S11

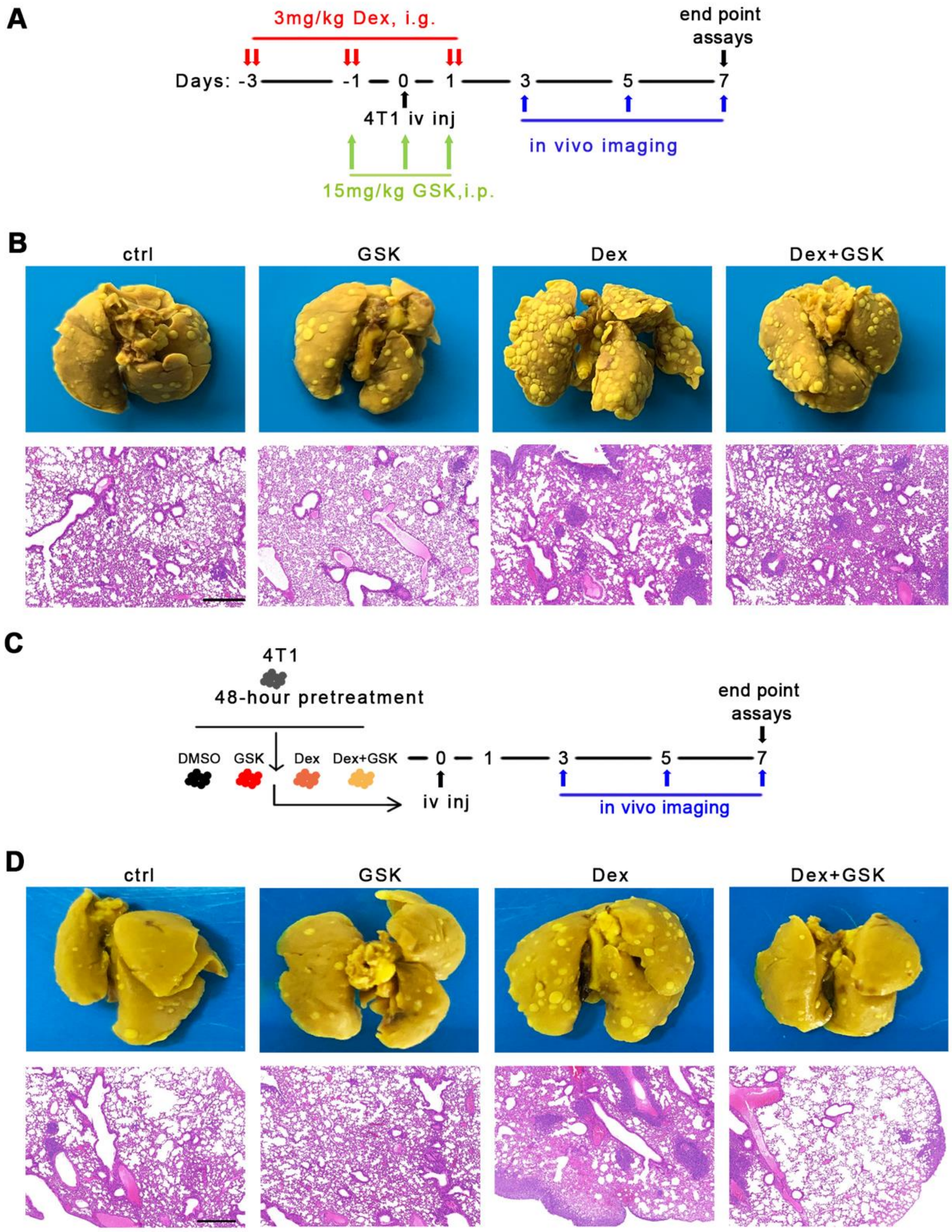

Figure S11. The effect of GSK650394 on metastasis in experiment lung metastatic model. (A) Treatment scheme in Figure 4H-I. (B) Typical mouse lung tissue fixed with Bouin's fixation and H&E staining of lung tissue. (C)Treatment scheme in Figure 4J-K. (D) Typical mouse lung tissue fixed with Bouin's fixation and H&E staining of lung tissue.

Figure S12

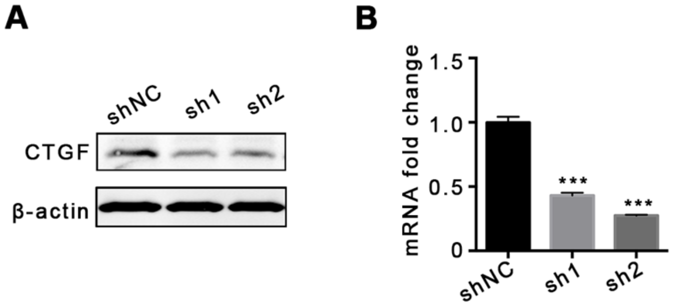

Figure S12. The efficacy of shRNA targeting CTGF in 4T1 cells. (A) Protein level of CTGF expression. (B) mRNA level of CTGF expression. n=3 replicates. Mean ± SEM is shown. ***p < 0.001.

Figure S13

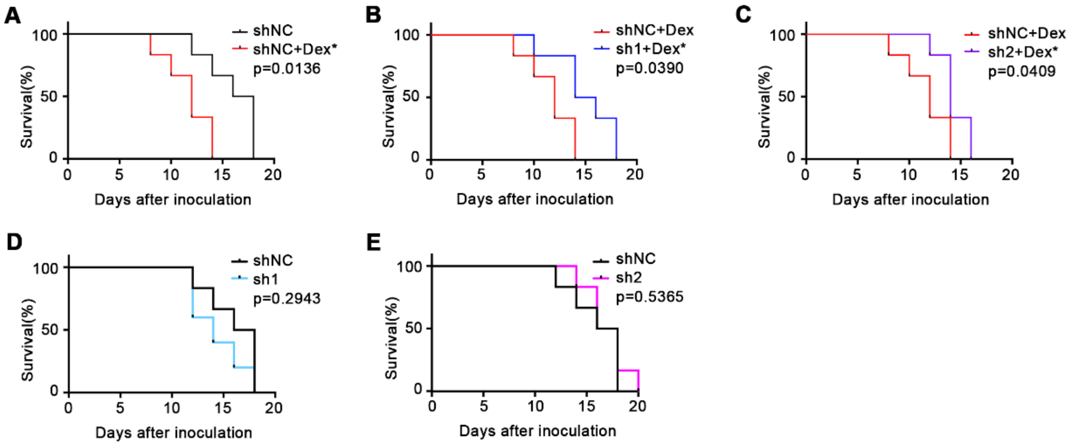

Figure S13. The survival of CTGF knockdown in 4T1 experiment lung metastasis. 4T1 cells were pre-treated with DMSO/100nM Dex for 48h, and then used for intravenous injection, n=6 mice per group (A) shNC vs shNC+Dex. (B) shNC+Dex vs sh1+Dex. (C) shNC+Dex vs sh2+Dex. (D) shNC VS sh1. (E) shNC vs sh2. *p < 0.05.

Supplementary Table 1. siRNA and gRNA sequences.

Name	Sequence
NC	5′- TTCTCCGAACGTGTCACGTAA -3′
CTGF#1	5′- CAAGCCTGTCAAGTTTGAGCTTTCT -3′
CTGF#2	5’-CACCAGTGTGAAGACATACAGGGCT-3’
CTGF#3	5’-GACATCTTTGAGTCCCTGTACTACA-3’
gRNA#1	5’- CTTCTTGAAAGTGATCGGAA-3’
gRNA#2	5’-GAACGATTTTATTCAGAAGA-3’
gRNA#3	5’-CCAACCCTCACGCCAAACCC-3’

Supplementary Table 2. Antibodies used for western blotting.

Name	Lot.	Company
anti-β-catenin	no.8480	Cell Signaling Technology
anti- N-cadherin	GB11109	Servicebio
anti- E-cadherin	no.14472	Cell Signaling Technology
anti- Vimentin	no.5741	Cell Signaling Technology
anti-SGK1	ET1610	Huabio
anti-CTGF	ER1802-69	Huabio
anti-PI3K	A0982	Abclonal
anti-p-PI3K	AP0854	Abclonal
anti-Nedd4l	A8085	Abclonal
anti-p-Nedd4l	no.12146	Cell Signaling Technology
anti-Smad2	no.5339	Cell Signaling Technology
anti-p-Smad2	no.18338	Cell Signaling Technology
anti-β-actin	Sc365062	Santa Cruz Biotechnology

Supplementary Table 3. Forward and reverse primers for RT-PCR.

Name	Sequence (5’-3’)
m-β-actin-F	ATATCGCTGCGCTGGTCG
m-β-actin-R	CGATGGAGGGGAATACAGCC
h-β-actin-F	TCAAGGCTGAGAACGGGAAG
h-β-actin-R	TCGCCCCACTTGATTTTGGA
m-SGK1-F	GCTCGAAGCACCCTTACCTA
m-SGK1-R	CATGCATAGGTGTTGCTGGC
h-SGK1-F	TTACTCCAGGATGAGGGGCA
h-SGK1-R	GGAGGAGAAGGGTTGGCATT
m-CTGF-F	AGAACTGTGTACGGAGCGTG
m-CTGF-R	GTGCACCATCTTTGGCAGTG
h-CTGF-F	AGGGCAAAAAGTGCATCCGT
h-CTGF-R	CTTCTTCATGACCYCGCCGT
m-Itga6-F	TGAAAGTCTCGTGCCCGTTC
m-Itga6-R	CCAGGCCTTCTCCGTCAAAT
m-Itgb1-F	TCGATCCTGTGACCCATTGC
m-Itgb1-R	AACAATTCCAGCAACCACGC
